# Feasibility and utility of Point-of-Care electronic clinical data capture in Uganda’s healthcare system: a qualitative study

**DOI:** 10.1093/jamia/ocad034

**Published:** 2023-03-08

**Authors:** Josephine Nabukenya, Andrew Alunyu Egwar, Lydia Drumright, Agnes Rwashana Semwanga, Simon Kasasa

**Affiliations:** Department of Information Systems, School of Computing and Informatics Technology, Makerere University, Kampala, Uganda; Department of Information Systems, School of Computing and Informatics Technology, Makerere University, Kampala, Uganda; Department of Medicine, University of Cambridge, Cambridge, UK; Department of Information Systems, School of Computing and Informatics Technology, Makerere University, Kampala, Uganda; Department of Epidemiology & Biostatistics, School of Public Health, Makerere University, Kampala, Uganda

**Keywords:** eHealth, electronic clinical data capture, feasibility, healthcare system, Point-of-Care

## Abstract

**Objective:**

This study aimed to assess Uganda’s readiness for implementing a national Point-of-Care (PoC) electronic clinical data capture platform that can function in near real-time.

**Methods:**

A qualitative, cross-sectional design was adopted to obtain a snapshot of Uganda’s eHealth system landscape with an aim to assess the readiness for implementing PoC platform. A purposive sampling strategy was used to select the study districts per region, health facilities per district, and participants per facility or district.

**Results:**

Nine facilitators were identified, including health worker motivation to serve the community, affirmative action on eHealth financing, improved integrating information and communication technology (ICT) infrastructure, Internet and electricity power connectivity, improved human resource skills and knowledge, the culture of sensitizing and training of stakeholders on eHealth interventions, the perceived value of the platform, health workers’ motivation to improve health data quality, interest to improve data use, and continuous improvement in the eHealth regulatory environment. Other suggestions entailed several requirements that must be met, including infrastructure, eHealth governance, human resources, as well as functional and data requirements.

**Discussion:**

Uganda, like other low-income countries, has adopted ICT to help solve some of its health system challenges. Although several challenges face eHealth implementations in Uganda, this study revealed facilitators that can be leveraged and requirements that, if met, would facilitate the successful implementation of a near real-time data capture platform capable of improving the country’s health outcomes.

**Conclusion:**

Other countries with eHealth implementations similar to those faced in Uganda can also leverage identified facilitators and address the stakeholders’ requirements.

## INTRODUCTION

Globally, the value of integrating information and communication technology (ICT) into health systems has been recognized; for instance, Health IT has transitioned from not only solely supporting efficiency in recording and transferring health information between providers to near real-time surveillance for faster identification of public health problems,^1^ but also to sophisticated decision support in healthcare settings based on AI algorithms.^2–7^ Similarly, the Uganda government has also embraced ICT as one of the critical tools to support its socio-economic transformation and improve the efficiency and effectiveness in delivering services. This is demonstrated in Uganda’s national policy and planning framework documents, including the *Uganda Vision 2040*^8^ and the *National Development Plan II*.^9^

The Uganda Ministry of Health (MoH) recognized the value of eHealth and developed a strategy to realize a national vision of eHealth adoption.^10^ Among the strategic areas is “… strengthening the national capacity to optimize the management and use of eHealth resources for better health outcomes,”^10^ from which several different eHealth interventions for data capture have been developed for Uganda. However, many of these are predominately based on proof-of-concept and remain in pilot phases^10^^,^^11^; as such, their success is yet to be realized by healthcare leaders/managers.

To avert this pilot phenomenon, implementers need to assess facilitators for the potential success of introducing eHealth technologies. Authors agree that such an assessment informs the long-range information services plan, guides the selection and implementation of technologies with the potential to succeed, modifies and guides eHealth implementations and integration efforts, and can potentially prevent costly interventions.^12–14^ Such an assessment is a factor for the successful implementation of eHealth. For example, doing an eHealth readiness assessment before implementing the innovation has been found to facilitate the change process enabling organizations to adopt and avoid failure.^13^ Also, readiness assessment helps to determine whether persons whom the technology is meant for will accept to use it or fail.^15^ Taherdoost^15^ states that a “great technology and application might be designed, … but if people do not get involved and do not use it, the project is failed.” Therefore, while, there is a clear interest in developing an exemplary national data capture platform for Uganda, it is critical to determine its eHealth “readiness.” Some studies have looked at cost-of-return of electronic health records (EHRs), but these do not reflect Uganda’s setting^16^^,^^17^; thus, it is important to conduct these analyses locally.

There is a larger body of literature around eHealth readiness and technology adoption,^18–26^ though few focus on EHR adoption in low-income settings.^27^ Studies examining eHealth adoption in low- and middle-income countries (LMICs) settings suggest that a single model may not be sufficient.^28^ Studies that have assessed the quality of technology/EHR adoption models and post-implementation data^29^ provide different perspectives than preimplementation information. Therefore, to support a greater understanding of the eHealth implementation environment, we used constructs from 3 technology adoption models^18–21^^,^^26^^,^^30^ (1) Technology-Organization-Environment (TOE) framework^19^ provided a bigger picture of internal and external factors of eHealth adoption and implementation. TOE helped us to explore the technology, organizational and environmental contexts that would support implementation of the eHealth PoC data capture platform in Uganda. (2) Diffusion of Innovation (DOI) Theory^18^ supported our understanding of driving factors in adoption, and (3) Technology Acceptance Model (TAM)^26^ provided the variables to assess the perceived usefulness and ease of use of the proposed PoC data capture platform for Uganda. Overall, the 3 theories informed our exploration of support (management, financial, and network externalities), optimism, motivations and perceived value of adoption, technology and skills, and environmental conditions, including policy and regulations for implementing a PoC in Uganda.

We note that this study is part of a larger research that sought to (1) identify barriers and facilitators for electronic healthcare data capture and near-real-time processing to support healthcare and public health nationally; (2) assess the feasibility of near real-time data capture and processing platform for Uganda, and (3) provide a ‘road map’ for the MoH to use in considering their plan for advancing eHealth provision across Uganda. Specifically, in this article, we report on the feasibility and utility of Point-of-Care (PoC) electronic clinical data capture for Uganda regarding facilitators and requirements for implementing such a platform for Uganda’s Healthcare System. Uganda has the potential to benefit from implementing a PoC system, as highlighted by some authors^31^^,^^32^ including; improved data quality, improved patient–clinician interactions, timely sharing of patient/health data among clinical practitioners, increased use of the data for planning purposes, and improved management of health information.

## METHODOLOGY

### Study design and area

The study adopted a descriptive cross-sectional design to obtain a snapshot of Uganda’s eHealth system and assess readiness for implementing a PoC electronic clinical data capture platform. In addition, qualitative methods were explicitly employed to understand the feasibility and utility of implementing the platform.

To ensure representation of different regions of Uganda that experience different burdens of our study diseases conditions (ie, malaria and HIV), we used random sampling for high, medium, and low burden districts of each condition in each region of Uganda. Table 1 shows the resultant choice of study districts.

**Table 1.** Study districts of Uganda

Region	Low incidence districts	Medium incidence districts	High incidence districts
**Central**	1) Mukono	1) Rakai	1) Kayunga
	2) Lyantonde	2) Kassanda	2) Butambala
	3) Masaka	3) Kalungu	3) Mityana
	Ex) Kampala		
**Western**	1) Ntungamo	1) Bunyangabu	1) Kamwenge
	2) Mbarara	2) Hoima	2) Kitagwenda
	3) Sheema	3) Rubirizi	3) Ibanda
**Eastern**	1) Kapchorwa	1) Manafwa	1) Kumi
	2) Mbale	2) Iganga	2) Busia
	3) Serere	3) Soroti	3) Jinja
**Northern**	1) Napak	1) Abim	1) Adjumani
	2) Karenga	2) Maracha	2) Moyo
	3) Nabilatuk	3) Nebbi	3) Agago

*Note*: Kampala was included because of the large number of healthcare facilities and the high population density.

Therefore, the study was conducted in selected healthcare facilities representing a mix of urban and rural healthcare settings from all 4 regions of Uganda: Central, Western, Eastern, and Northern.

### Sample size, sampling strategy, and rationale for inclusion in the study

The study focused on a range of study populations that differed for interviews, focus groups, and key informant interviews, as shown in Table 2.

**Table 2.** Participant sampling strategy and reasons for including each category in the study

Participant category	Reason for inclusion in the study	Sample size	Sampling strategy	Data collection method
Government officials who work in a health-related capacity	To provide an understanding of the health information needs, potential impacts of near real-time reporting, and challenges of near real-time reporting.	60	Purposive and convenience sampling	Key informant interview
Representatives of commercial ICT-related companies in Uganda	To understand costs and partnerships that facilitate the implementation of near real-time reporting platforms in Uganda.	30	Purposive convenience sampling	Key informant interview
Health partners and non-government organizations (NGOs) involved in health provision and public health	To understand electronic health data capture systems already in place, the needs of the Country’s partners in health and interests/vision for future data capture.	20	Purposive and convenience sampling	Key informant interview
Health researchers	To discuss what electronic data they already capture, how these might fit into the system that we are developing, and what data and/or services they might find as beneficial for their work.	60	Purposive and convenience sampling	Key informant interview
Community Health Workers (CHEWs)/Village Health Teams (VHTs)	To discuss (1) facilitators and barriers to near real-time data capture and (2) what information they would like, in what form to support their work, should such a system become available.	200	Purposive and convenience sampling	Focus group
Health Professionals	To identify facilitators and barriers to near real-time data capture and suggest solutions like what information they would like, in what form to support their work.	200	Convenience and quota sampling	Interviews
Adult members of the general Uganda population	To elicit a wide set of population views on appropriate use of individuals’ health data on the proposed platform.	160	Purposive and convenience sampling	Focus group

Participants included healthcare workers selected from different healthcare facilities within the 4 regions of Uganda. Other participants were drawn from commercial partner organizations, non-government organizations (NGOs), research institutions, government entities, and ICT professional organizations.

### Data collection and analysis

In preparing this article, we followed guidelines for reporting qualitative research in informatics^33^ as seen in the subsections that follow.

#### Data collection

Data were collected through interviews and focus group discussions (FGDs) using the telephone or Zoom platform. Owing to their merit in gathering rich information about the phenomenon of study, interviews and focus groups were preferred to support eliciting experiences and views of potential users regards the barriers, facilitators, and expectations for a PoC data capture platform. The interview was semi-structured with questions framed to allow flexibility but based on concepts from the technology adoption theories^18^^,^^19^^,^^21^; which aimed at assessing driving factors of technology adoption, internal and external factors of eHealth adoption and implementation, and usefulness and ease-of-use of a technology. The focus groups supported deeper understanding of the facilitators and barriers to near real-time data capture, information that users of a PoC platform could expect to support their work, and views on appropriate use of personal health data.

Research assistants and study investigators did the data collection. To ensure consistency in data collection, the research assistants were recruited, trained, and pre-tested the data collection tools (interview and focus group guides). During data collection, the responses were all audio-recorded and transcribed verbatim.

#### Data analysis and interpretation

Data analysis involved importing the transcripts into Nvivo version 11. Analysis was undertaken using the thematic approach (Supplementary Appendix SA) by coding the datasets. Coding first involved a small set of transcripts by the 3 members of the analytical team. These were discussed to identify key codes of interest that would be used on the rest of the transcripts. The output of the coding of themes is in Supplementary Appendix SA. Coding of both interviews, and FGDs transcripts helped to triangulate data from the 2 sources. Coding of the transcripts supported members of the analysis team in identifying emerging themes that were eventually merged to form the main themes. The coders compared, discussed, and agreed on themes where they differed. Thematic analysis was preferred to analyze the qualitative data collected to generate themes inductively. Emerging information was synthesized to indicate Uganda’s readiness to implement and use the PoC electronic clinical data capture platform.

To ensure trustworthiness in the course of this research, 4 quality measures of *credibility*, *transferability*, *dependability*, and *confirmability*^34^ were addressed. *Credibility*: prolonged engagement with participants (between 30 and 45 min for interviews sessions and 60–90 min for focus group sessions) permitted the data collectors to probe responses to elicit credible information. Furthermore, participants were permitted to use examples to elaborate their responses. Methodological triangulation was used by gathering data through interviews and focus groups. Also, investigator triangulation was applied by recruiting several researcher assistants for data collection, and using 4 researchers to analyze and interpret the data. The transcripts were individually analyzed by 3 researchers and the interpretations compared. Where the interpretations differed, the analysis team discussed and reached consensus on a suitable interpretation. The use of the different datasets supported data triangulation. *Transferability* in qualitative research requires detail description of the participants and the research process.^34^ To support our readers to assess whether the study findings are transferable to their setting, we have reported in “Participants’ distribution” section, the characteristics of the participants categories, their qualifications, and relevant work experiences. The aspects of consistency (*dependability*) and neutrality (*confirmability*) are concerned with analysis and interpretation of the data. Two researchers who were not part of the data analysis and interpretation reviewed the outputs from the data analysis team against the original transcripts to ensure that no vital information was left out due to subjectivity of the analysis team.

### Ethical considerations

Ethical approval was sought from Uganda National Council for Science and Technology (HS1005ES). Participants consented by filling out forms attached to the set of interview questions. In addition, those study participants who answered telephone interviews provided verbal consent before the actual start. All data gathered from this study were stored confidentially.

## RESULTS

### Participants’ distribution

One hundred ninety-five individuals participated in the study; 169 were interview participants; and 33 participated in 31 FGDs. Most participants were healthcare professionals drawn from health facilities who completed semi-structured interviews (49%), 5% were CHEWs/VHTs FGDs, 3% were community FGDs, 9% were government organizations, 9% were Health partners/NGOs, 13% were commercial partner organizations, and 12% were researchers. The FGDs were conducted among Community Health Workers/Village Health Teams (CHEWs/VHTs), with each group comprising 4–7 participants. Whereas each interview lasted between 25 and 30 min, FDGs took between 45 and 90 min. Table 3 shows the characteristics of the participants in this study.

**Table 3.** Background characteristics of the participants

Healthcare professionals		Researchers		Government institutions	
Characteristic	%	Characteristic	%	Characteristic	%
**Gender**		**Gender**		**Gender**	
Male	46.8	Male	56.0	Male	64.7
Female	52.0	Female	44.0	Female	35.3
Did not indicate	1.2				
**Age group**		**Age group**		**Age group**	
21–30	31.3	Less than 41	20.8	21–30	8.8
31–40	42.5	41–50	54.2	31–40	44.1
41–50	19.4	51+	25.0	41–50	38.2
51+	6.0			51–60	8.8
N/A	0.8	**Highest education level**			
**Professional years worked**		Master’s degree	52.1	**Years worked**	
Under 1	6.7	Doctorate degree	47.9	Less than 5	11.8
2–5	30.0	**Year degree was received**		5–10	73.5
6–9	24.2	Before 2012 (10 years & above)	15.2	11+	14.7
10–13	17.8	2012–2016 (5–9 years)	34.8		
14–17	9.1	2017+ (0–4 years)	50.0		
18+	11.5				
**CHEWs/VHTs**		**Commercial partners**		**Health partners/NGOs**	
**Characteristics**	**%**	**Characteristics**	**%**	**Characteristics**	**%**
**Gender**		**Gender**		**Gender**	
Male	42.3	Male	90.2	Male	61.1
Female	57.7	Female	9.8	Female	38.9
**Age group**		**Age group**		**Age group**	
21–30	11.9	21–30	25.5	21–30	2.8
31–40	26.8	31–40	35.3	31–40	25.0
41–50	33.9	41–50	27.4	41–50	55.6
51–60	19.6	51–60	11.8	51–60	16.7
61–70	7.7	**Years worked in the company**		**Years worked in the organization**	
**Years worked as CHEW**		Less than 5	24.0	Less than 1 year	8.6
Less than 5	78.3	5–10	56.0	1–5	20.0
5+	21.7	11+	20.0	6–10	42.9
**Years worked as VHT**				11+	28.6
1–5	15.0				
6–10	41.8				
11–15	11.8				
16–20	19.0				
21+	12.4				

Overall, the majority of the respondents were males in all categories of respondents. The age groups with the highest response range from 32 to 40 for healthcare professionals, commercial partners, and government institutions; and 41–50 for CHEWs/VHTs, researchers, and Health Partners. Most of the participants from the healthcare professionals, CHEWs, VHTs, commercial partners, government institutions, and Health Partners/NGOs categories had worked 5–10 years with the organizations/institutions. Only the researcher’s category presented participants with the least years of work experience (0–5) years.

### Feasibility for near real-time electronic data capture platform for Uganda

The feasibility of implementing a near real-time electronic clinical data capture platform for Uganda lies partially in mitigating barriers to data collection and sharing, health data access and use, obtaining high-quality research data, and implementing electronic medical records (EMRs)/(EHRs).

#### Facilitation of health workers and their motivation to serve the community

Health workers are motivated to continue providing care to their communities through financial rewards. Although some reported that their motivations included government and NGO support in terms of drugs and equipment, seeing patients recovering from illness, training, and salary, others argued that there were no facilitators … “*facilitation of our work; monthly salary, meetings with health facilities and giving allowances for work done. We would ensure provision of books for recording, mobile phones to ease communication, transport means to quicken service delivery*” (KyFG_01, CHEW/VHTs Focus Group #11).

#### Positive intention to finance eHealth environment

Although health financing remains one of the biggest challenges in LMICs, participants recognized government and donor communities’ efforts to financially support the eHealth environment … “*MoH have budgets that have been allocated to support these [eHealth] systems. Largely supported by donors*” (HPO_03, Health Partner/NGOs Interview). Furthermore, the participant further acknowledged that donors provide funding for several health activities, for example, HIV and immunization data can never be missed to be reported because of the money attached to it, right from data collection to data cleaning.

#### Improved ICT infrastructure including internet and electricity power

Uganda has several electronic data capture systems implementations, some of which have overlapping functionalities. For example, UgandaEMR manages HIV and TB clients and is not spread across all health facilities; DHIS2 is used to collect health data from all health facilities; mTRAC is used for weekly surveillance reports where facilities do submit them by SMS, and the biostatistician offices receive the records on a dashboard. Some health workers use their laptops, which might pose a risk in terms of data security: “*…We don’t have computers or modems; I use my laptop to do my work. The district data is stored on my laptop, and I use my modem to access the Internet*” (HPKD, Health Professionals Interview).

Also, there is improved network coverage and widespread availability of electric power sources that can be used to charge electronic devices “…*the implementation of this system is possible in Uganda because there is electricity in many parts of the country and there is widespread accessibility (though it is still expensive)*” (HIR_01, Health Informatics Researcher); “*Mobile telephone coverage and broadband services also enable people at any point to collect and share data through the Internet over available platforms. Where the Internet is not fast enough, there is mobile telephony, e.g., SMS, USSD platforms, mobile voice, etc. Despite the challenges that come with network reliability when collecting and sharing health data, the infrastructure is now available*” (UCC_02, Government Organization Interview). Participants confessed that a large number of health facilities had broadband services that support data collection.

#### Improved ICT technical skills and knowledge set to build and manage the system

Uganda has continued to train IT professionals and software developers. These are facilitators of the eHealth domain, where they provide the technical support required to develop, implement and maintain eHealth systems. One respondent stated, “*As a country, we now have a good ICT capacity. So, developing such a system would not be a big challenge because we already have the technical skills developed from within the country. So, it is possible to implement this system in Uganda, and the other thing is that people are now embracing ICT everywhere due to this COVID-19; everything is going online*” (HIR_04, Health Informatics Researcher Interview).

#### Culture of sensitizing and training stakeholders on healthcare interventions

Participants argued for sensitization of the community on how the collected data should be used, its benefits and training on how to use the electronic devices. Participants felt that key personnel are often trained based on project goals and that users are always equipped with skills and guidelines of what needs to be done. Participants further believe that project implementation may fail due to resistance from the community where such a project will be implemented. In some cases, local politicians may sabotage certain government programs due to political differences; hence, community awareness has to be done.

#### Perceived system value

In terms of value for the system to Uganda’s health system, respondents believed that this would improve the decision-making process because there would be timely reporting and electronic storage of data. Such a system will advance technology, and once a technology is in place, there will be improved dynamics of how work will be done. Likewise, the system will come with a business sense since a lot of money will be saved with reduced paperwork and time spent in capturing data. The respondents also believe such a system is a worthwhile investment in Uganda.

#### Determination to improve data collection, quality, and ownership

Participants reported several efforts made to improve electronic data collection. These efforts can be leveraged to facilitate Uganda’s envisioned PoC electronic data capture platform … “*We have efforts that have gone a long way in facilitating data collection and use. We have had a number of trainings to health workers, their utilization of HMIS tools, for example, we have a lot of support by Bio-stat to ensure that we all have this data coming in*” (HPO_03, Health Partner/NGOs Interview). When asked about the feasibility of an electronic data capture platform for Uganda, one respondent said; “*I think it is possible, right now people are getting used to collecting data on phones, tablets, I think it is picking up, it is convenient, it is quick of course; the challenge is when the data is sent to the central system, can they be able to retrieve it or access it? It looks like you enter the data into the system then the data collector may not be able to access it*” (HIR_05, Health Informatics Researcher Interview). However, participants also expressed doubts about the ownership and ease of access to such data, which may affect usage, especially in supporting timely patient care. Participants reported increasing demand for quality data to account for service delivery and monitor progress in relation to the input. They argue that data standardization is very important, without which data becomes meaningless if everyone interprets it differently.

#### Increased interest in improving data-driven decision-making

Participants reported interest by senior management and donors in using health data to inform their decision-making processes … “*The Minister and Permanent Secretary are always in the news talking about perinatal death … Senior management is always asking for those numbers and tracking how these numbers go down … donors like Global fund, GAVI who are very much in need of data … if you look at HIV data it’s the most analyzed and used*” (HPO_03, Health Partner/NGOs Interview); “*The data are analyzed for donor reporting or if Senior management has an interest in certain issues like maternal health, Covid-19 and the like*” (HPO_03, Health Partner/NGOs Interview). Therefore, the need or importance of the data facilitates data collection.

#### Improved policy and regulatory environment

Uganda developed a national eHealth strategy that points to key regulations for the eHealth environment, including laws, governance structure, and standards. Most of these are still under development. However, the current health governance structure can be leveraged to support the implementation of such a PoC electronic data capture platform. Governance and leadership structures also enable data generation at all levels. A respondent said, “*We have also contributed to a number of reforms, especially as far as legal and regulatory frameworks are concerned, to ensure that data that is coming in is put into use. In terms of guidelines and SOPs, a number of policy reforms and strategies have been developed… Ideally, this [will] foster reporting and use of the data*” (HPO_03, Health Partner/NGOs Interview). Besides, the health implementation environment is used to deploy and adhere to stringent guidelines. Currently, their practices include meeting stringent timelines when partners set reporting timelines that must be adhered to. MoH sets deadlines within which data should be entered into the system, the requirement that a facility should have someone responsible for health data management, and should implement electronic tools such as mTrac, DHIS2, etc.

### Requirements for implementing an electronic data capture platform

The participants shared challenges they experienced while using existing siloed systems to deliver healthcare services to Uganda and provided suggestions to address such challenges. Participants’ suggestions may be considered requirements for implementing Uganda’s PoC electronic data capture platform, grouped into technical infrastructure, governance, workforce, functional, and data requirements.

#### Technical infrastructure required for a timely model data capture system

The ICT infrastructure in health facilities is below the minimum required level to support a national PoC electronic data capture platform. For example, “*the Internet in some hospitals may be found in particular offices like laboratories and administrators’ offices. This follows earlier drives made to digitise health services, which realised sub-optimal results, leading to donor and government fatigue at the health facility level*” (NITAU_03, Government Organizations Interview). Currently, some health facilities have no computers, electricity, stable power, network coverage, alternative power source, data coverage, and one or 2 solar panels … “*Largely, we are still at paper-based data collection at the health facility. Some few computers exist which are used to track commodities, drugs that have been given out, consumables, HIV and OPD*” (HPMF, Health Professional Interview). A minimum level of investment is needed to benefit from ICTs, in which the government and implementing partners are willing to re-invest. Health facilities must be connected to the national internet grid to harness a national PoC electronic data capture platform, as observed by a health partner; “*A number of facilities are still out of network reach with poor internet connectivity to foster reporting*” (HPO_04, Health Partner/NGOs Interview).

#### Governance of eHealth infrastructure

##### Absence of documented SOPs and guidelines

The MoH should develop documented guidelines and procedures to access data and information systems through its governance structures, including the Division of Health Information and Steering Committees. Currently, the challenge is that the procedures are not documented. Worse still, the frameworks, such as the data access framework, are not clear enough to be operationalized. “*We have guidelines, for example, data process framework, but the kind of understanding in the operationalisation of this is not very clear*” (HPO_01, Health Partner/NGOs Interview).

One health respondent proposed some of the contents to be considered in the SOPs, and respondent HPO_04 added that any identifying information should not be granted to anyone requesting access to the data … “*They should state the variables that they want so that they can answer their objectives, and those responsible for giving data should make sure that the data they are giving should ensure that the data is not easily identified so that they cannot trace the data to the given individual who has health issues*” (HPO_04, Health Partner/NGOs Interview).

The MoH should also be supported in regulating health service points at the lower levels since the health industry is complex and extensive. “*… supporting the Ministry’s role in regulating these organisations and ensuring that systems that are contributing to patient data at whatever service point end up at MoH coordination*” (HPO_01, Health Partner/NGOs Interview).

##### Political will

Health Development Partners financially support health data services in the country and are uncoordinated to a larger extent. Yet the resources to facilitate evidence-based programming and decision-making are a hindrance, as mentioned by a health development partner. “*At the national level, we have the resources to create the environment that allows evidence-based programming. So, the budget is very minimal to support this and it largely supported by partners, therefore, a hindrance*” (HPO_01, Health Partner/NGOs Interview).

##### Uncoordinated donor efforts

Fragmented/siloed information systems result from uncoordinated donor efforts; as one health partner mentioned, “*We have a challenge of fragmentation of systems that are not even interoperable. You have these many donors coming on board, but government finds itself in a position where every donor is contributing. Still, they are not well coordinated in the efforts of steering committees. We still have a gap because we don’t have standards yet. Everyone develops a system, and this piloted system is operating in many fragmented systems*” (HPO_01, Health Partner/NGOs Interview).

##### Capacity building of health workers on ethics

Health workers need to be trained on ethics of data collection to maintain the integrity of collected data and the quality of the analyzed data … “*there is an issue of the workforce, if you don’t train your data collectors well, they don’t have the right ethics. Somebody will sit down under a tree and make-up data … from a statistical standpoint, if you have poor quality data, the results are going to be as good as the data that you put in*” (HIR_08, Health Informatics Researcher Interview).

##### Data sharing guidelines

Well-documented SOPs can assist with ethical data access. Additionally, the people requesting data must clearly state their objectives and the required variables. There’s a need to anonymize critical data so that it cannot be traced back to the owners. Finally, there should be guidelines for sharing clinical data with agreements that can be enforced. Under all circumstances, the benefits should outweigh the risks mentioned by a respondent working in a government HIV program. “*People need to be able to sign agreements that they are not going to do wrong staff with data. Those principles cannot be compromised*” (HIV_03, Government Organization Interview).

##### Responsibility for data access and regulation

Respondents agreed that the MoH should be responsible for providing oversight for data collected in real-time from healthcare and public health providers in Uganda. Specifically, the respondents from health partner institutions said; “*Access is primarily supposed to be Ministry of Health*” (HPO_01, Health Partner/NGOs Interview); “*There can be a body or a department somewhere in the Ministry of Health where it could be responsible for the data or trust and confidentiality issues*” (HPO_04, Health Partner/NGOs Interview); “*the Division of Health Information should regulate access and have clear SOPs that document the process one must go through to access national health data*” (NITAU_02, Government Organization Interview).

#### Human resource capacities

##### Inadequate staffing at health facilities

Health facilities should have data collection responsibilities as core to their functions. Health workers collect data in real-time, with the health facility responsible for the data collected. However, health facilities are understaffed, and thus health workers cannot simultaneously perform dual roles of data collection and service provision. Digitization could partly solve the under-staffing challenge as mentioned by a Health partner; “*many clinicians do administrative work ideally needing some support. But if the system is digitalised, they don’t have to run a lot of administrative data because it would give them summary reports and everything automatically. So, collection at the time of input, the health facility in charge would be responsible*” (HPO_03, Health Partner/NGOs Interview). Therefore, the Ministry should digitize hospital information systems to reduce the burden of making summaries and lessen administrative work on clinicians.

##### Quality of human resources

The human resources handling data at health facility and community levels are not skilled enough to manage data in its rightful dimensions. One health partner mentioned that the situation is even worsened at the community level by CHEWs/VHTs … “*Even before we brought in technology, their capacity in terms of paying attention, recording the correct data, and dimensions of quality were a challenge. It is even worse at the community level. You find a community health worker who is supposed to be reporting quarterly but is not able to write. Then when you bring in technology, it becomes worse*” (HPO_01, Health Partner/NGOs Interview).

#### Functional and data requirements

##### Processed data

Data that are usually available in systems is not processed, giving health partners the additional duty of cleaning it before being used. “*We normally get it in a raw form, and we are unable to make sense out of it, but our kind of desire would be getting data in a more usable form, like when I go to the system and simply look at the data where a lot of cleaning has been done., a lot of processes have been done*” (HPO_01, Health Partner/NGOs Interview).

##### Data with GPS coordinates

Data received from communities with GPS coordinates enables geospatial analysis. It also allows health workers to locate patients who need health interventions … “*We always need more data around GPS coordinates for communities that we are serving, and we kind of make geo-social analysis*” (HPO_01, Health Partner/NGOs Interview).

##### Bidirectional data sharing

There is a need for health data to be shared vertically, that is, data collectors with health managers and vice versa. Whereas managers need the information for planning, collectors and other lower-level health system stakeholders may need it for health education. Some participants from FGDs expressed the need for bidirectional information flow … “*We have counts of diseases in our registers which we summarise in our reports and help us to track the causes of such big numbers and the possible interventions that we can do locally and jointly after reporting to the healthcare facility, we also use these studies to compare with other fellow VHTs in other communities and copy each other on the best practices they are using*.” (NeFG_01, CHEW/VHTs Focus Group #17); “*We need statistics of health happenings in our communities*” (HFG_02, CHEW/VHTs Focus Group #6); “*We would like to receive this information through training workshops and presenting summaries of the information collected so that we could do community mobilisation, sensitisation, and health education*” (IFG_01, CHEW/VHTs Focus Group #3).

##### Simple data/information sharing formats

Finally, FGDs revealed that lower-level health system stakeholders expect information shared to be presented in simple, easy-to-understand formats and details. Suggestions were made for pie charts, simple words or pictures and of comparative nature whenever necessary … “*In a pictorial form but in a language, I understand, they can draw graphs or charts or summarise them this would help me to teach it to the community*” (KyFG_02, CHEW/VHTs Focus Group #12); “…*picture data makes a lot of sense in programming … such data in that format is used in making a case or sense of whatever we are reporting about*” HPO_01; “…. *comparisons, we need to know if our work is helping our community or not*” (AFG_01, CHEW/VHTs Focus Group #1).

## DISCUSSION

### Feasibility

Results reveal 9 facilitators for a near real-time PoC electronic clinical data capture platform for Uganda. Some of the facilitators are similar to those identified in developing countries/LMICs,^35–38^ that led to the successful implementation of eHealth systems. As opposed to classifying the facilitators as patient-level facilitators and others as clinician-level facilitators,^35^ this study used the TOE^19^ framework to classify the facilitators into technology, organizational, and environmental facilitators. Whereas each of the 9 facilitators (ie, health worker financial and personal motivation to serve the community, affirmative moves by government and donor community to finance the eHealth environment, improved ICT infrastructure including Internet and electricity power supply and enabling environment, improved ICT technical skills and knowledge set to build and manage the eHealth system, culture of sensitizing and training of stakeholders on healthcare interventions, perceived value of the system among health workers, need to improve health data collection, quality, and ownership, increased interest to improve data-driven decision-making, and continuously improving policy and regulatory environment) can be classified at clinician-level or patient level, they are of a technology, organization and environment nature.

There’s a need to motivate everyone involved in the data chain, right from the person who is the lowest of the chain, especially where data capture starts to senior management at the MoH.

Our study findings show general positive attitude exhibited by health workers and management, which corroborates the argument that healthcare work requires a positive attitude toward work.^38^ Our study findings evidence that improved financing by management, financial facilitation, and framing of policy guidelines to regulate the eHealth environment are all positive facilitators, that in turn improves health worker motivation to work. In fact authors suggest that results are great whenever health workers have a positive perception/opinion of an intervention and are motivated to work.^38^^,^^39^ Both financial and non-financial motivators are important in improving work; from the findings, we claim that if Uganda can create an environment of balanced internal (personal perception/opinion) and external (financial and non-financial) motivators for healthcare providers, then proper implementation and use of the PoC platform would be supported.^36^

The study reported improved ICT infrastructure, Internet and connection of health facilities to the national electrical power grid. These are infrastructure resources that are facilitators for deploying a digital platform including the proposed PoC eHealth data capture platform.^40–42^ These, coupled with other factors such as availability of funds and ICT technical support team, will ensure the platform is serviced and continually maintained to ensure the eHealth initiative function and scale.^40^

This study also found out that there is improvement in ICT training among health workers in Uganda, which has potential to promote the adoption to using existing eHealth technologies. Actually, training in using a technology/platform is considered a facilitator for implementing eHealth technology.^36^^,^^42^ Therefore, to further reduce potential complacency levels to use eHealth platform as claimed to be common among health workers with lower eHealth literacy,^42^ there should be stakeholder commitment to train health workers. Such training should be based upon appropriate training curriculum/program for both in-service health workers and pre-service health professionals. However, it is not clear from literature that such a curriculum or systematic plan for training of healthcare workers exists for Uganda. We argue that only a systematic plan will ensure consistent and inclusive country-wide training of all health workers.

Regarding improved policy and regulatory environment as another facilitator of PoC platform, it is clear that Uganda has progressed in the development of eHealth policy and strategy^10^^,^^43^ to guide eHealth development. Similar facilitators have been identified in other studies’ platforms.^35^^,^^36^ For example, in the eHealth strategic plan,^10^ Uganda planned to solve the fragmentation and lack of interoperability^11^ by standardizing the eHealth environment. The decision to develop contextual standards for Uganda was prompted by the need to solve the problem of lack of interoperability that exists among several eHealth interventions in the country.^11^ The Interoperability problem has been reported in several studies as endemic and hampering data exchange, use and utilization.^11^^,^^44^ Therefore, to date, Uganda is progressing with the development of standards and an enterprise architecture framework to support existing ICT laws and regulations like the data protection and privacy act of the electronic environment.^43^

We argue that each facilitator contributes to the establishment of an enabling environment for developing, implementing, using, and maintaining such a PoC electronic clinical data capture platform. Therefore, Uganda should exploit the strengths to build an eHealth platform capable of supporting improved health services delivery to its population.

### Requirements

Despite the development of ICT and the Internet, which has provided access as a platform for data sharing and reuse among interested stakeholders, the study findings show that there’re still some challenges in implementing such a platform in Uganda. Some of the challenges to data use may include; integrating data from various sources, lack of data management professionals, and lack of proper understanding of the data, among others.^45^ However, in developing countries, data sharing offers numerous benefits beyond national interests and is for the benefit of all mankind.^46^ Some respondents in this study argued that there is a need for data sharing for sustainable development. Pastorino et al^45^ concur that data sharing can improve patient outcomes and evidence-based healthcare decision-making. Therefore, Uganda needs to create an enabling environment for data sharing and use. For example, through the development of integrable eHealth systems/platforms that support timely sharing of health data, operationalization of relevant eHealth policies, standards and regulations that relate to health data sharing and use, and training of data management staff and potential users to ensure proper appreciation of health data, requisite security measures, and privacy use.

The study findings further revealed that some health workers at the community level could not even write correctly. This status quo affects not only the quality of paper-based data collection but may be worse when using eHealth technology. Actually, it has been shown that low literacy levels negatively affect health workers’ attitudes toward using electronic platforms to collect health data.^47^ The study findings that showed challenges with health worker capacity gaps at the health facility and community level are similar to Miseda et al’s^48^ study that highlighted overall skills gaps in 46 counties. To bridge the gap, our study participants recommended training all staff involved in data management. Training of health workers have been shown to improve data management practices.^49^ Beck et al^50^ affirm that addressing knowledge/skills gap in data security require training of new staff and updating all staff on relevant procedures for ensuring safeguards of data during its entire life cycle. This is mainly true in LMICs, where increasing health data/information is being collected to monitor and evaluate the use, cost, outcome, and impact of health services.^50^ Actually, in confirming the training as a major remedy to the skills gap as recommended by Miseda et al,^48^ this study advocates that both in-service training and updating of staff are required to improve health workers attitude toward eHealth systems^47^ and data management practices.^49^

Another critical requirement considered for implementing such a platform for Uganda is data governance. Uganda should have a health system wide framework for managing health data throughout its lifecycle. The framework should balance the 2 needs of collecting and securing the data while getting value from the information derived therein. Therefore, as recommended by Rosenbaum,^51^ Uganda’s data governance framework should establish (1) policies for access, management, and permissible uses of data, (2) data stewardship methods and procedures, and (3) qualifications data users and their data access conditions. In addition, it should supervise health data management, sharing and include its use for patient care purposes.^52^ Strengthening Uganda’s current data governance structures will enable them to use the framework to solve identified governance challenges. For example, the eHealth data governance body will ensure relevant SOPs and guidelines for health data management are developed, coordinate management of data being handled by donor supported Health interventions in the country, and supervise compliance with health data security and privacy regulations among others.

Finally, we argue that though evidence of the impact of facilitators was not investigated, their steady progression will support attainment of some of the requirements for implementing the PoC data capture platform for Uganda. Therefore, based on this study, we claim that the identified facilitators are sufficient to support the implementation of a near real-time data capture platform for Uganda.

## CONCLUSION

The study sought to identify facilitators and requirements for implementing PoC electronic clinical data capture for Uganda’s Healthcare System. A possible limitation of the study could have been appropriately synthesizing the huge volume of qualitative data. However, the results of the analysis showed that Uganda possesses 9 facilitating factors that present a conducive environment for implementing such a platform; accordingly, Uganda is nearly ready to implement this platform that can function in near real-time. However, there are gaps still existing in the eHealth environment that the country must continue to address, expressed as requirements for implementing a near real-time electronic clinical data capture platform for Uganda. Uganda needs to further improve and maintain the technical infrastructure required to support such a PoC platform, police the implementation environment to ensure compliance with set eHealth laws and regulations, continue to build the human resources capacity of the staff that operate the PoC platform, and supervise the quality and use of eHealth data that the platform will capture.

## Supplementary Material

ocad034_Supplementary_Data

## Data Availability

The data underlying this article cannot be shared for ethical and privacy reasons.
