# Supplementary material for: Feasibility and utility of Point-of-Care electronic clinical data capture in Uganda’s healthcare system: a qualitative study
Source: J Am Med Inform Assoc. 2023 Mar 8;30(5):932–42. doi: 10.1093/jamia/ocad034 (PMC10114114; doi:10.1093/jamia/ocad034)
Supplement: ocad034_Supplementary_Data [file ocad034_supplementary_data.docx]

Feasibility and Utility of Point-of-Care Electronic Clinical Data Capture in Uganda’s Healthcare System: A Qualitative Study

**Corresponding author**

**Josephine Nabukenya**

Department of Information Systems, School of Computing and Informatics Technology, Makerere University, Kampala, Uganda

PO Box 7062, Kampala, Uganda

Email: [josephine@cit.ac.ug](mailto:josephine@cit.ac.ug)

Telephone: +256 776 658800

**Other Authors**

**Andrew Alunyu Egwar**

Department of Information Systems, School of Computing and Informatics Technology, Makerere University, Kampala, Uganda

**Lydia Drumright**

Department of Medicine, University of Cambridge, Cambridge, United Kingdom

**Agnes Rwashana Semwanga**

Department of Information Systems, School of Computing and Informatics Technology, Makerere University, Kampala, Uganda

**Simon Kasasa**

Department of Epidemiology & Biostatistics, School of Public Health, Makerere University, Kampala, Uganda

**Keywords**: eHealth, Electronic Clinical Data Capture, Feasibility, Healthcare System, Point-of-Care

**Word count: 6686**
